# Bilirubin Ameliorates Oleic and Palmitic Acid Accumulation in an In Vitro Model of MASLD

**DOI:** 10.33549/physiolres.935696

**Published:** 2026-02-01

**Authors:** Kateřina POSPİŠILOVÁ, Nikola TULACHOVÁ, Jakub ONHAJZER, Aleš DVOŘÁK, Libor VİTEK

**Affiliations:** 1 Institute of Medical Biochemistry and Laboratory Diagnostics, First Faculty of Medicine, Charles University and General University Hospital in Prague, Prague, Czech Republic; 2 Department of Biochemistry and Microbiology, University of Chemistry and Technology, Prague, Czech Republic; 3 Laboratory of Cell and Developmental Biology, Institute of Molecular Genetics of the Czech Academy of Sciences, Prague, Czech Republic; 4 Fourth Department of Internal Medicine, First Faculty of Medicine, Charles University and General University Hospital in Prague, Prague, Czech Republic

**Keywords:** Bilirubin, Fatty acids, Fenofibrate, MASLD, PPARα

## Abstract

Metabolic dysfunction-associated steatotic liver disease (MASLD) is a chronic progressive disorder characterized by an excess accumulation of lipids in the liver. The aim of this study was to examine the role of bilirubin (BR), the catabolic heme product and a putative peroxisome proliferator-activated receptor α (PPARα) agonist, in an *in vitro* model of MASLD. In our study, we used human hepatoblastoma HepG2 cells exposed to oleic (OA)/palmitic acid (PA) (2:1 ratio, 24 h) with subsequent treatment with BR or fenofibrate (FF, a clinically used PPARα agonist) at clinically relevant concentrations. A significant increase in total cellular lipid content after OA/PA treatment (*p*<0.05) was observed. When treated with BR and FF, intracellular concentrations of OA and PA decreased significantly (*p*<0.05). Changes in lipid content were attenuated by GW6471 (a PPARα antagonist) indicating the importance of PPARα pathway in a mechanism of action of BR and FF. Furthermore, we observed a significant increase in the gene expression of a pyruvate dehydrogenase kinase 4 after treatment with FF; FF also increased mitochondrial respiration. Collectively, our data indicate that both BR and FF reduce the accumulation of OA/PA in HepG2 cells exposed to these fatty acids, presumably by up-regulating fatty acid oxidation *via* PPARα pathway.

## Introduction

Metabolic dysfunction-associated steatotic liver disease (MASLD) is a chronic liver disorder characterized by an excessive accumulation of fat in hepatocytes, in the absence of significant alcohol consumption or other secondary causes of liver steatosis [1]. In recent years, MASLD has been increasingly recognized as the liver manifestation of metabolic syndrome, closely related to obesity, type 2 diabetes mellitus (T2DM), dyslipidemia, and insulin resistance [2]. The pathogenesis of MASLD is often described by the “multiple hit” hypothesis [3], where several insults act simultaneously: metabolic factors (such as insulin resistance, hormones secreted from adipose tissue, nutritional factors, gut microbiota) [4], as well as genetic and epigenetic factors [5]. The initial phase, characterized by simple steatosis, involves an excessive accumulation of triacylglycerols within hepatocytes. This is mainly due to an imbalance between the lipid acquisition (*via* increased free fatty acid (FFA) uptake and *de novo* lipogenesis [6]) and the lipid disposal (*via* fatty acid oxidation and their export in the form of very low-density lipoproteins [7]). Over time, this lipid overload can lead to lipotoxicity, oxidative stress, mitochondrial dysfunction, and subsequent activation of inflammatory pathways, contributing to the progression of simple steatosis to steatohepatitis [3], fibrosis, cirrhosis, and ultimately hepatocellular cancer [8]. Its widespread prevalence (more than 25 %) [2] has significant public health implications, as MASLD not only increases the risk of liver-related morbidity and mortality but also contributes to an elevated risk of cardiovascular disease (CVD), which is the leading cause of death in these patients [9]. Despite its high prevalence and associated health risks, there are currently only few pharmacological treatment options for patients with MASLD, underscoring the urgent need for effective therapeutic strategies [10].

Bilirubin (BR), traditionally considered a waste product of heme catabolism, has recently emerged as a potent signaling molecule with a wide range of physiological functions, including antioxidant, anti-inflammatory and cytoprotective effects [11]. Epidemiological studies have consistently shown that subjects with mildly elevated systemic concentrations of unconjugated BR (Gilbert’s syndrome) are at a lower risk of developing a spectrum of civilization diseases such as CVD [12], T2DM and metabolic syndrome [13], autoimmune diseases, and certain types of cancer [14]. Not surprisingly, a similar protective role has also been observed in patients with MASLD [15]. Interestingly, this balance is reciprocal – lower serum BR concentrations are associated with obesity, dyslipidemia, CVD, and T2DM [16].

These findings could be partially attributed to the potent antioxidant and immunomodulatory functions of BR [17], but in recent years, BR has also been recognized as a metabolic hormone that drives gene transcription as an agonist of various cellular receptors, such as the constitutive androstane receptor (CAR), the pregnane X receptor (PXR) and, most importantly, the peroxisome proliferator-activated receptor α (PPARα) [11].

The nuclear receptor PPARα, also called the ‘master regulator’ of liver lipid metabolism, enhances fatty acid oxidation by upregulating genes involved in both fatty acid transport, β-oxidation and ketogenesis [18]. Simultaneously, PPARα modulates insulin sensitivity, reduces liver glucose production (inhibiting glycolysis), and promotes gluconeogenesis, which helps to maintain blood glucose levels during fasting [19]. Therefore, it is not surprising that the role of PPARα has been intensively investigated in the context of the pathogenesis of MASLD and as a promising therapeutic target [20].

Recent experimental studies have shown that BR can activate PPARα in mice, leading to lower blood glucose concentrations, increased fatty acid oxidation, and reduced lipid accumulation in the liver [21]. Simultaneously, treatment of 3T3-L1 adipocytes with biliverdin (precursor of BR) suppressed lipid accumulation and upregulated PPARα target genes [21,22]. Furthermore, BR has been shown to improve insulin sensitivity and reduce oxidative stress, further supporting its potential role in the treatment of MASLD [23]. In one of our previous *in vitro* studies, elevated BR concentrations upregulated PPARα downstream effectors of PPARα such as FGF21 (fibroblast growth factor 21) and ANGPTL4 (angiopoietin-like 4) [24]. The beneficiary effect of BR was also observed in white adipose tissue, where BR activated lipolytic pathways mediated by PPARα in diet-induced obese mice with mild hyperbilirubinemia [22]. These potential therapeutic effects have been confirmed in the experimental animal model, in which mice administered BR nanoparticles improved steatosis and reduced fat cell size mediated *via* PPARα [25].

In addition to animal studies, the “*in vitro* MASLD” models are widely used to investigate the potential benefits of various experimental therapeutics and chemopreventive compounds, including pharmaceuticals [26] or nutraceuticals such as dietary polyphenols [27]. The most widely used *in vitro* model is a human hepatoblastoma cell line (HepG2) exposed to a mixture of oleic acid (OA) and palmitic acid (PA). While OA is considered more steatotic, PA exerts pro-oxidant effects. To mimic chronic liver steatosis with less apoptotic and toxic effects, the 2:1 ratio (OA:PA) has been generally recommended [28].

Therefore, the objective of our study was to assess the potential beneficiary effects of BR in this *in vitro* model of MASLD, and to compare these effects with fenofibrate (FF), a PPARα agonist clinically used as a hypolipidemic drug [29]. We specifically focused on the effects of BR on lipid metabolism and on determining whether these effects are indeed mediated through PPARα activation.

## Methods

### Overall design

As an *in vitro* model of MASLD, we used HepG2 cells exposed to OA/PA with subsequent treatment with BR or FF at clinically relevant concentrations. We a) analyzed the cytotoxicity of OA/PA as well as BR and FF; b) determined the accumulation of lipids by staining the cells with a Nile red fluorescence dye; c) quantified intracellular fatty acid concentrations using GC/MS; d) analyzed the mRNA and protein expressions of key modulators of lipid metabolism; and e) measured mitochondrial respiration using high-resolution respirometry.

### Chemicals

All chemicals and reagents were obtained from Merck (Germany) unless otherwise specified. Commercial BR was purified as previously described [30]. Aliquots of stock solutions of BR (2.5 and 5 mmol/l), FF (25 and 50 mmol/l) and GW6471, a PPARα antagonist, (7.5 mmol/l) were prepared in sterile dimethyl sulfoxide (DMSO), stored at −20 °C and diluted 1,000 times (BR and FF) or 750 times (GW6471) directly in culture medium before use.

### Cell culture

HepG2 cells (ATCC, VA, USA) were cultured in phenol red-free Dulbecco’s Modified Eagle Medium (DMEM; IMG CAS, Czech Republic) supplemented with 5 mmol/l glucose, 2 mmol/l L-glutamine, 1 % non-essential amino acids (Biosera, France) and 10 % fetal bovine serum (FBS; Biosera, France). Culture conditions were normoxic 5 % CO_2_ chamber at 37 °C in a humidified atmosphere. For experiments, cells were seeded on appropriate plates for two days (on the day of the experiment, the cell culture typically reached an approximate confluency of 80 %).

### Preparation of FFA solutions

FFA-bovine serum albumin (BSA) solutions were prepared as previously described with a 5:1 (FFA:BSA) ratio [31]. Briefly, OA and PA were dissolved in absolute ethanol (Penta, Czech Republic) and then diluted 300 times in pre-warmed culture medium supplemented with 0.4 mmol/l FFA-free BSA to reach the final concentration of OA or PA of 2 mmol/l. Culture media with FFA-free BSA and the corresponding amount of absolute ethanol served as a control solution. Binding of FFA with BSA was carried out overnight on a shaker at 37 °C and 5 % CO_2_. The following day, the stocks solutions were sterile filtered, aliquoted and kept at −20 °C until further use. Prior to use, these solutions were directly diluted into the culture medium.

### Cell treatment

Following the initial 48-hour culture period, cells were exposed to OA/PA (2/1 v/v) or control solution for 24 h (stock solutions were diluted 10 or 4 times to reach final concentrations of 200 or 500 μmol/l, respectively). In some experiments, PPARα inhibition was implemented between steatosis induction and treatment with BR/FF: fresh medium with GW6471 (final concentration 10 μmol/l or DMSO (1.5 μl/ml; negative control) was added for 2 h. Subsequently, the medium was replaced, and the control and OA/PA cell samples were incubated with BR (2.5 and 5 μmol/l), FF (25 and 50 μmol/l) or DMSO (1 μl/ml; negative control) for additional 24 h. The final concentrations of Bf (bilirubin free, unbound, biologically active fraction of BR) [32] in the culture media corresponded to non-toxic, mildly elevated BR concentrations [24] and the final concentrations of FF were based on the known plasma levels of patients treated with FF [33].

### Cytotoxicity

For the assessment of changes in viability due to treatment, the MTT (3-(4,5-di methyl thiazol-2-yl)-2,5-diphenyltetrazolium bromide) assay was used. The cells were seeded in 96-well plates and after treatment, the medium was replaced with the culture medium containing MTT (1 mg/ml) and incubated for 15 min. Cells were lyzed with DMSO and the signal was read at 570 nm (TECAN Infinite M200 spectrophotometer, Sweden). The bandwidth for absorbance measurements was 5 nm. The results were compared with those for wells with relevant control solutions.

### Neutral lipid staining

Neutral lipid accumulation was quantified after staining with a Nile red fluorescence dye. After treatment, cells seeded in 96-well plates (UV transparent well flat clear; Corning Incorporated, NY, USA) were incubated in culture medium with a dye (1 μg/ml) for 10 min. Fluorescence (excitation/emission: 515/585 nm) was quantified on a fluorescence spectrophotometer (TECAN Infinite M200 spectrophotometer, Sweden) in fresh medium without a Nile red dye. The bandwidths for excitation and emission were 5 and 20 nm, respectively. The results were compared with those for wells with relevant control solutions.

### Fluorescence microscopy

Nile red was also used to visualize the accumulation of lipids with fluorescence microscopy. Cells were seeded in 12-well plates (UV transparent well flat clear; Corning Incorporated, NY, USA) with 18 mm coverslips coated with a poly-L-ornithine solution. After induction and treatment, cells were incubated with a Nile red dye as described above, washed twice with PBS and fixed in 4 % paraformaldehyde for 15 min. The coverslips were mounted with Vectashield mounting medium (with DAPI (4′,6-diamidino-2-phenylindole) fluorescent stain (Vector Laboratories, Inc, NY, USA) and imaged on a fluorescence microscope at 63× magnification (Leica DMI-6000, Germany. Illumination: HXP 120W/45C VIS Hg lamp – Leica EL6000 for fluorescence, objective: HCX PL APO 63×/1.40 OIL; FWD 0.14; CG 0.17 | BF, POL, PH. Filtercubes DAPI (A) (Ex: 360/40; DM 400; Em: LP 425) and TRITC (N3) (Ex: 546/12; DM 565; Em: 600/40), monochromatic sCMOS camera Leica DFC 9000; 6,5 μm pixel, QE: min. 82 %. Software: LAS X).

### GC/MS analysis of intracellular fatty acid concentration

OA, PA, stearic (STEA) and linoleic (LIN) acids were quantified by GC/MS, with a modified method described previously [34]. Cell samples were washed with ice cold PBS, lyzed, and extracted with water/methanol/chloroform (1:1:2, v/v/v) with the addition of an internal standard (IS, margaric acid) and centrifuged at 1,000× g for 10 min. The lower non-polar phase was transferred to glass vials, dried under nitrogen gas, and the neutral lipids were hydrolyzed with KOH. FFA were extracted with ethanol/hexane (2:1, v/v) after neutralization with acetic acid. The upper hexane phase was dried under nitrogen gas and derivatized with diazomethane (room temperature, 20 min). The samples were dried again, dissolved in hexane and injected directly into a GC/MS (7000D GC/TQ/MS; GC column HP-5MS UI; 30 m×0.250 mm×0.25 μm, Agilent Technologies, CA, USA). The amount of analyte was normalized with IS to the protein content in cell pellets. The results were compared with wells with relevant control solutions.

### Quantitative Real-Time polymerase chain reaction (PCR)

Pyruvate dehydrogenase kinase isoform 4 (PDK4) and liver isoform of carnitine palmitoyltransferase 1 (CPT1A) mRNA levels were evaluated using quantitative real-time PCR previously described [24]. In brief, total RNA was extracted using a GenUP Total RNA Kit (BiotechRabbit, Germany), and complementary DNA (cDNA) was synthesized with a High Capacity cDNA Reverse Transcription Kit (Applied Biosystems, CA, United States). Amplification of the target genes was performed on a ViiA 7 instrument (Applied Biosystems) in 10-μl reaction volumes, containing 4.5 μl of 10-fold diluted cDNA template from a completed reverse transcription reaction, TaqMan Advanced Master Mix (Applied Biosystems) and TaqMan™ Gene™Fast Expression Assay (Hs00912671_m1 *CPT1A*, Hs01037712_m1 *PDK4*, Hs00947536_m1 *PPARα*, Hs02800695_m1 human HPRT, Applied Biosystems). The temperature profile was: 2′ 50 °C, 92 °C 10′ 40× (1″ 95 °C, 20″ 60 °C). The relative quantification was made by the 2^−ΔΔCt^ method with *HPRT* as a housekeeping gene. The results were compared to wells with relevant control solutions. RNA quality control was performed spectrophotometrically using Nanodrop (DS-11+ Spectrophotometer, DeNOVIX Inc., DE, United States).

### Western blot

Protein levels of PPARα were analyzed by Western blotting. Cell samples were washed with ice-cold PBS and extracted with a lysis buffer (5 M NaCl, 1 M Tris, pH=8, 10 % Triton-X 100) with the addition of proteinase and phosphatase inhibitors, thoroughly vortexed and incubated for 30 min on ice. The samples were centrifuged (15 min at 15,000× g, 4 °C), the required amount of supernatant (35–40 μg of protein) was diluted with loading buffer (4× Laemli Sample buffer, Bio-Rad Laboratories, CA, USA), denatured at 95 °C for 10 min and separated by SDS-PAGE electrophoresis (4–20 % GenScript Sure PageTM system). Proteins were transferred to a nitrocellulose membrane, blocked in EveryBlot Blocking Buffer (Bio-Rad Laboratories, CA, USA) for 10 min and then incubated with primary antibodies: mouse monoclonal anti PPARα (Santa Cruz Biotechnology, 1:1,000 v/v dilution) and rabbit polyclonal anti β-actin (dilution 1:1,000 v/v) (Cell Signalling, MA, USA) for 1 h at room temperature. After washing in TTBS buffer, the membranes were incubated with swine anti-rabbit IgG-HRP secondary antibody (β-actin) (Dako, Glostrup, Denmark) or anti-mouse IgG-HRP (PPAR-α) and visualized with SuperSignalTM West Femto Maximum Sensitivity Substrate (Thermo Scientific, MA, USA). Further analysis was performed using ImageJ 1.54k software. The results were compared to those of the wells with relevant control solutions.

### High-resolution respirometry

The mitochondrial oxygen consumption in living cells was measured as previously described [24]. Briefly, cells were harvested with trypsin, centrifuged for 5 min at 500× g, and the pellet was resuspended in 2.5 ml of fresh medium. Routine respiration and electron transfer (ET) capacity (uncoupled state after carbonyl cyanide-p-trifluoromethoxyphenylhydrazone (FCCP) titration) were measured on Oxygraph-2k (Oroboros Instruments GmbH, Austria) in a 2 ml chamber in culture medium. Respiratory parameters per cell were analyzed in Datlab 7.4.0.4 software (Oroboros Instruments, Austria) and compared with those cells with relevant control solutions.

### Statistical analyses

Data were tested for normal distribution and are expressed as mean ± SD or median with interquartile range (Western blot results). Differences between variables were evaluated using the Student’s unpaired *t*-test with Welsch correction or Mann-Whitney Rank Sum test depending on data normality, and were considered statistically significant at *p*<0.05. Statistical analyses were performed with Prism 10.4.0 software (GraphPad, CA, USA).

## Results

### Induction of steatosis in HepG2 cells

**Fig. 1:**
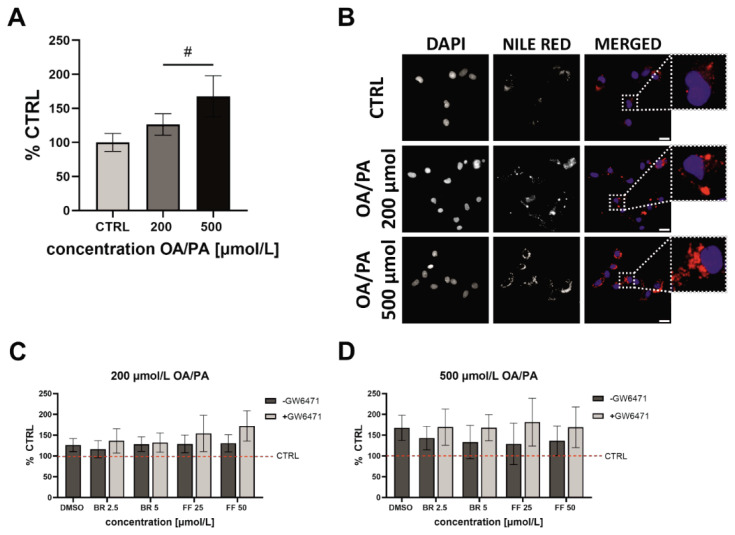
Neutral lipid content in HepG2 cells after exposure to OA/PA (200 or 500 μmol/l (**A** and **B**)) and subsequent GW6471 and BR or FF treatment (**C and D**). The neutral lipid content after exposure of HepG2 cells to fatty acids as assessed with Nile red for neutral lipid quantification (**A**) or visualized with fluorescent microscopy (**B**). Steatotic cells ((**C**) 200 mmol/l; (**D**) 500 mmol/l) were first treated with PPARα antagonist GW6471, then with BR (bilirubin) or FF (fenofibrate). **^#^** *p*<0.05 compared to control cells (CTRL), n=18 for experiments without inhibitor and n=12 for experiments with inhibitor. Scale: 25 μm. CTRL: control, untreated cells unexposed to FFA; DMSO: treated cells exposed to FFA. OA, oleic acid, PA, palmitic acid.

Compared to untreated control HepG2 cells, the neutral lipid content, a marker of cell steatosis, increased significantly after exposure to fatty acids (by 26 % and 68 % after exposure to 200 and 500 μmol/l of fatty acids, respectively, *p*<0.05, Fig. 1a). An increased lipid content after exposure to a higher concentration of OA/PA was also markedly visible when using fluorescent microscopy (the number and size of the lipid droplets increased, Fig. 1b). Neither treatment with BR nor with FF had any effect on the lipid content in steatotic cells (200 μmol/l, Fig. 1c, d). Pretreatment of steatotic cells with PPARα antagonist GW7641 had some elevating effect on the neutral lipid content in both concentration models, but also here; the differences did not reach statistical significance (Fig. 1c, d).

### The effects of FFA exposure and BR and FF on HepG2 cell viability

**Fig. 2:**
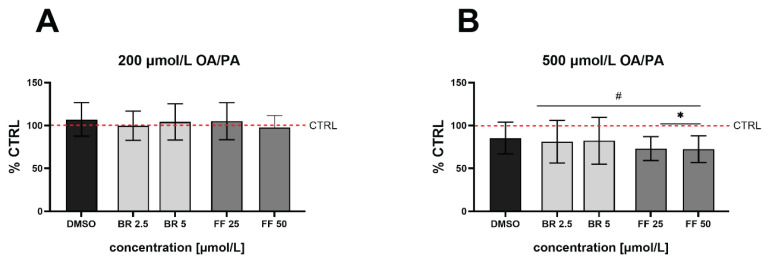
The effect of OA/PA and BR/FF on HepG2 cell viability. Steatosis was induced by exposure of cells to OA/PA (200 μmol/l, (**A**), 500 μmol/l (**B**)) and subsequently treated with BR (bilirubin) or FF (fenofibrate). **^#^** *p*<0.05 compared to control cells, * *p*<0.05 compared to DMSO cells; n=30 each experiment. CTRL: untreated cells unexposed to FFA; DMSO: treated cells exposed to FFA.

Changes in cell viability were measured using the MTT test after induction of steatosis (with 200 and 500 μmol/l concentrations of OA/PA) and subsequent treatment with BR and FF (2.5 and 5 μmol/l, and 25 and 50 μmol/l, respectively). No toxic effects were observed when using lower concentrations of fatty acids for induction of steatosis, as well as when subsequently treated with BR or FF (Fig. 2a). However, when exposed to a higher concentration of OA/PA, cell viability was reduced following treatment with BR and FF (*p*<0.05, Fig. 2b).

### Intracellular concentration of fatty acids in HepG2 cells exposed to FFA and treated with BR and FF

**Fig. 3:**
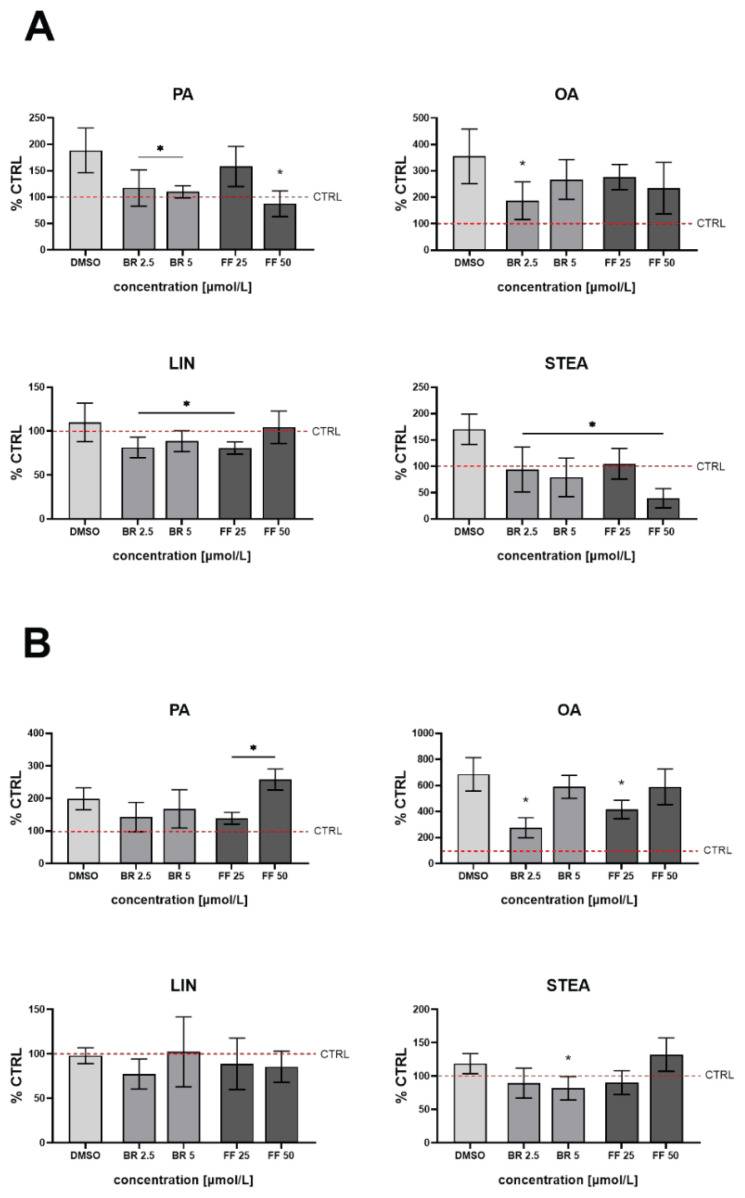
Intracellular concentrations of selected FFA in HepG2 cells exposed to 200 μmol/l (**A**) or 500 μmol/l (**B**) of OA/PA and treated with BR and FF. Intracellular concentrations of OA (oleic acid), PA (palmitic acid), LIN (linoleic acid) and STE (stearic acid) acid were analyzed using GC/MS after induction of steatosis and treatment with BR (bilirubin) and FF (feno-fibrate). **^#^** *p*<0.05 compared to control cells, * *p*<0.05 compared to DMSO cells, n=6 for each experiment. CTRL: untreated cells unexposed to FFA; DMSO: treated cells exposed to FFA.

To further analyze the effect of BR and FF on changes in the intracellular lipid profile in HepG2 cells exposed to FFA, the intracellular concentrations of selected fatty acids were measured using GC/MS. In addition to the OA and PA used for the induction of steatosis, the intracellular concentrations of STEA and LIN were also quantified. As expected, exposure of HepG2 cells to OA and PA led to massive accumulation of both fatty acids in cells. When exposed to 200 μmol/l of OA/PA, the concentrations increased in exposed HepG2 cells from 1.04±0.5 to 3.28±0.7 μg/mg of the cell protein for OA (*p*<0.001, Fig. 3a), and from 6.67±2.6 to 11.24±3.6 μg/mg of the cell protein for PA (*p*<0.005), whereas concentrations of STEA and LIN were much lower and almost comparable (3.42±2.2 vs. 4.4±1.6 μg/mg for STEA, and 0.2±0.1 vs. 0.28±0.1 μg/mg for LIN, *p*>0.05 for both comparisons) (Fig. 3a). When exposed to 500 μmol/l of OA/PA, the concentrations of both fatty acids in exposed HepG2 cells increased further (Fig. 3a), while the concentrations of STEA and LIN were still much lower and almost comparable (Fig. 3a).

Compared to control cells, the intracellular concentration of PA decreased significantly by more than 1.7-fold after both BR treatments and by more than 2-fold after treatment with a higher dose of FF, respectively (*p*<0.05, Fig. 3a). In the case of OA, whose concentration increased by more than 3.5-fold (OA/PA), a significant decrease was observed after treatment with a lower BR concentration (by 2.7-fold, *p*<0.05, Fig. 3a). Steatotic cells retained the same concentration of LIN, which then decreased significantly after treatment with BR/FF (with the exception of FF 50 μmol/l) (by 1.29-fold, 1.21-fold and 1.29-fold after both BR treatments and FF 25 μmol/l, respectively, *p*<0.05, Fig. 3a). And finally, the concentration of STEA, which increased by 1.7-fold due to exposure to OA/PA (200 μmol/l), significantly decreased after treatment with both BR and FF (by 1.76-fold, 1.91-fold, 1.65-fold and 2.3-fold after BR and FF treatments, respectively, *p*<0.05, Fig. 3a).

When 500 μmol/l OA/PA were used, the decrease in PA concentration after treatment with BR/FF was not significant; however, treatment with FF 50 μmol/l resulted in a significant increase (by 1.59-fold *p*<0.05, Fig. 3b). The intracellular concentration of OA increased more than 6-fold, but a much smaller increase was observed after treatment with lower concentrations of BR and FF (by 5.1-fold and 3.7-fold, respectively, p<0.05). Again, exposure to OA/PA did not change intracellular concentration of LIN, and none of the BR and FF treatments had any effect on intracellular concentration of this fatty acid (Fig. 3b). A less pronounced beneficiary effect of BR and FF treatments was also observed for intracellular STEA concentration (Fig. 3b).

### The effect of BR and FF on PPARα expression in control and steatotic HepG2 cells

**Fig. 4:**
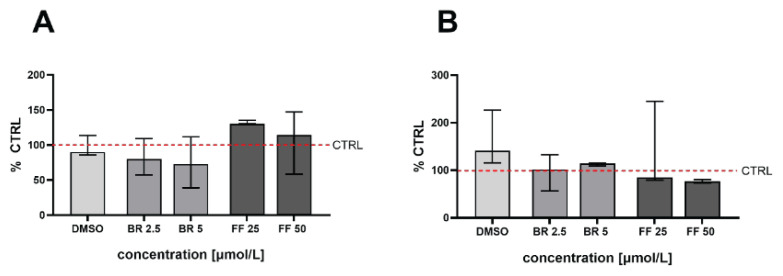
PPARα protein expression in HepG2 cells exposed to 200 μmol/l (**A**) and 500 μmol/l (**B**). PPARα protein expression was analyzed using Western blot after steatosis induction and BR (bilirubin) and FF (fenofibrate) treatment (n=3 for each experiment). DMSO: treated cells exposed to FFA. Data expressed as median and IQ range of % control.

Exposure of HepG2 cells to OA/PA (both 200 and 500 μmol/l) did not affect *PPARα* gene expression (data not shown), nor the PPARα protein expression was significantly changed (Fig. 4a–b).

### The effect of BR and FF on PDK4 and CPT1A gene expression in control and steatotic HepG2 cells

**Fig. 5:**
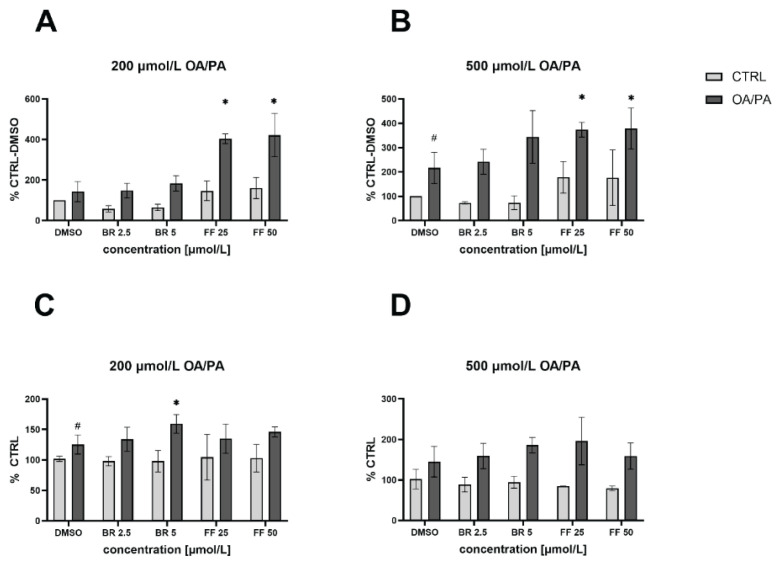
The effect of BR and FF on *PDK4* (**A**) and *CPT1* (**B**) gene expressions in steatotic HepG2 cells. BR: bilirubin; FF: fenofibrate; *PDK4*: mitochondrial pyruvate dehydrogenase lipoamide kinase isozyme 4; *CPT1*: liver isoform of carnitine palmitoyltransferase 1. **^#^** *p*<0.05 compared to CTRL-DMSO cells, * *p*<0.05 compared to OA/PA-DMSO cells, n=4 for each experiment n=4 for each experiment. CTRL: untreated cells unexposed to FFA.

To closely examine the mechanism of the observed lipid lowering effects of BR and FF in steatotic HepG2 cells, the expression of two genes involved in fatty acid metabolism (*PDK4* and *CPT1*) was determined. Since activation of *PDK4* is associated with the switch to fatty acids as the main energy fuel [35], its OA/PA concentration-dependent up-regulation was also observed in steatotic cells (Fig. 5a). Significant up-regulation was observed after exposure to OA/PA (200 μmol/l) and subsequent treatments with FF (by 261 and 279 %, respectively, *p*<0.05, Fig. 5a). When challenged with OA/PA (500 μmol/l), DMSO-treated cells significantly increased their expression of *PDK4* (by 117 %, *p*<0.05, Fig. 5a). Treatment with fatty acids and FF further increased mRNA levels by 157 % and 162 %, respectively (*p*<0.05, Fig. 5a).

Like *PDK4*, the up-regulation of *CPT1A* indicates a higher rate of β-oxidation of FFA, which is due to its role in the transfer of fatty acids to the mitochondrial matrix [35]. In accord with the up-regulation of *PDK4*, mRNA levels of *CPT1A* were positively associated with OA/PA exposure and further significantly increased after treatment with BR 5 μmol/l (combination of OA/PA 200 μmol/l exposure and BR 5 μmol/l, by 34 %, *p*<0.05, Fig. 5b).

### The effect of BR and FF on mitochondrial respiration of steatotic HepG2 cells

**Fig. 6:**
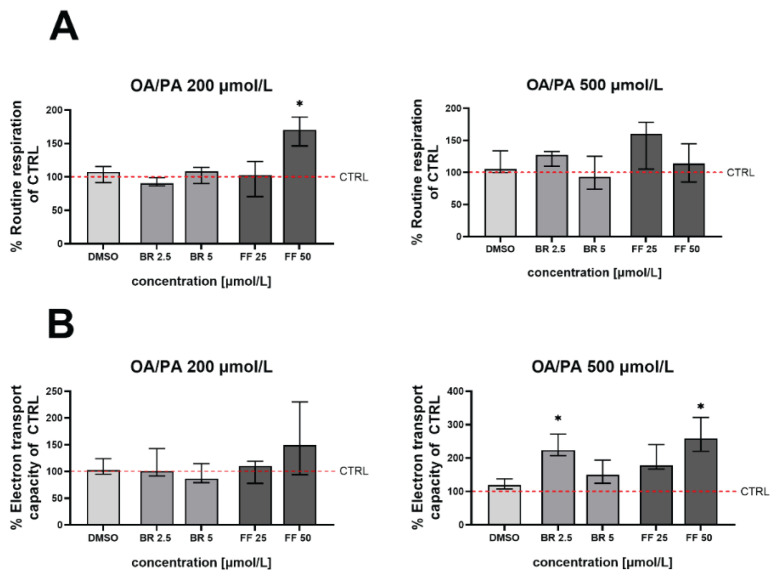
Routine respiration (**A**) and electron transport capacity (**B**) in steatotic HepG2 cells treated with BR and FF. Routine respiration and electron transfer capacity (uncoupled state after FCCP) were measured in living cells using high-resolution respirometry after steatosis induced by exposure to OA/PA (200 and 500 μmol/l) followed by treatment with BR (bilirubin) or FF (fenofibrate). * *p*<0.05 compared to DMSO, n=5 for each experiment. DMSO: DMSO treated cells exposed to FFA. CTRL: control cells unexposed to FFA.

To evaluate the effect of BR and FF on the mitochondrial activity of steatotic HepG2 cells, their routine respiration and electron transfer capacity were measured. With the exception of FF (50 μmol/l after exposure to OA/PA 200 μmol/l, Fig. 6a), no significant effect on routine respiration was observed (Fig. 6a); however, when exposed to a higher concentration of fatty acids, this effect reversed. An opposite trend was observed in electron transfer capacity (Fig. 6b).

## Discussion

During the past century, our knowledge of the role of BR in a human body has changed significantly – from a neurotoxic waste product of heme catabolism, through discoveries of its profound antioxidant and anti-inflammatory effects, and finally to recognition of BR as a crucial endogenous molecule with various metabolic and signaling functions with a potentially profound impact on human health [11,16]. Recent evidence has suggested that BR (as well as its precursor biliverdin) directly induces gene response *via* its binding to the nuclear receptor PPARα [11,21], a crucial regulator of energy homeostasis, therefore acting as a metabolic hormone [11]. Biliverdin treatments significantly reduced lipid accumulation in 3T3-L1 white adipose tissue cell model and significantly increased expression of mitochondrial and lipid burning genes such [21,22]. Indeed, there are many epidemiological studies that report an association of mildly elevated BR concentrations with lower cardiometabolic risk factors [17]. Such observations may partially account for the interaction with PPARα mentioned above [21].

In our study, we used a HepG2 cell line, a well-established *in vitro* model of MASLD [26]. Furthermore, the transcriptome responsiveness of the same HepG2 cells to BR was shown to be predominantly mediated by PPARα [36]. Although the protocol used with a controlled FFA:BSA ratio of 5:1 has been recommended for the development of steatosis rather than for lipotoxicity [31], we observed a slight decrease in viability of steatotic cells challenged with 500 μmol/l OA/PA, most likely due to lipotoxicity, which was further emphasized in cells treated with FF. On the contrary, treatment with a lower concentration of OA/PA did not alter viability, but also did not markedly change the number and size of intracellular lipid droplets, an important marker that differentiates between healthy and affected hepatocytes [37]. This relatively low increase in intracellular lipid content may explain why subsequent treatment did not produce any observable differences among the experimental groups. To eliminate potential confounding effect of lipotoxicity exerted by higher concentrations of OA/PA, we quantified (per μg of cell protein) intracellular concentrations of OA and PA that increased abundantly in exposed cells, together with STEA and LIN. Remarkably, treatment with BR decreased the intracellular concentrations of selected fatty acids (OA, PA, STEA, and LINO) even in steatotic cells challenged with a lower concentration of OA/PA. These results indicate that BR can effectively reduce the content of specific fatty acids, in particular those used for the induction of steatosis (OA/PA). This is in agreement with the results of experimental [21] as well as epidemiological studies [17].

The trend in different response to inhibition of PPARα between BR and FF effects could be due to the broader metabolic effects of BR compared to FF. Parallel to PPARα activation, BR is also a ligand transactivator of CAR and PXR [38], xenobiotic-sensing nuclear receptors and also important metabolic regulators. Both receptors have been reported to directly modulate gluconeogenesis and interact with nuclear receptors that regulate lipogenesis [39]. Importantly, CAR activation ameliorated liver steatosis in obese mice [40], further extending the possible mechanism of action of BR.

In our study, up-regulation of *PDK4* expression followed OA/PA induction was demonstrated, apparently because PDK4 acts as a metabolic switch, changing cellular energy preferences from glucose to fatty acid utilization [35]. These data are in agreement with an increase in the *CPT1A* mRNA expression previously reported in another *in vitro* MASLD study [27]. Increased levels of *PDK4* and *CPT1A* indicate that treatment of steatotic cells with BR/FF causes a change in the substrates utilized by mitochondria. Since routine respiration was not evaluated in permeabilized cells, to allow external addition of ADP^+^, ATP-synthetase saturation probably represented a rate-limiting step. In our studies, only FF caused a significant increase in routine respiration, which is consistent with a recent observation of an uncoupler-like effect of FF in adipose tissue [41]. Interestingly, this effect was lost when cells were challenged with a higher concentration of OA/PA, which could indicate that another rate-limiting step is the transport of fatty acids by CPT1A [42]. Interestingly, after exposure to higher concentrations of OA/PA and BR 2.5 or FF 50 treatment, the ET capacity was significantly enhanced, suggesting possible uncoupling properties of BR and FF. However, we cannot exclude that an observed increase ET capacity can be due to increased mitogenesis resulting in augmented glycolytic and lipolytic capacity.

To closely examine whether fatty oxidation was enhanced, a protocol utilizing permeabilized cells would be required. The role of mitochondria in the pathogenesis and progression of MASLD remains a subject of debate. Although some studies reported an increase [43], other studies documented a decrease in lipid oxidation [44]. This inconsistency probably reflects the dynamic role of these organelles in the pathogenesis of MASLD, which seems to depend on the stage of the disease [45].

Our data suggest that both BR and FF have the potential to alter intracellular lipid content. However, there are certain limitations to our study. First, *in vitro* models of MASLD exhibit considerable variability, and this has also been apparent in our study. Furthermore, data from *in vitro* models cannot be translated directly into human pathogenesis. To address other issues, the lipid-lowering effects of BR *in vitro* were demonstrated primarily in adipocytes [21,22] and in a transcriptomic study in the HepG2 cell line, treatment involved a high concentration of biliverdin, rather than BR. These methodological differences make direct comparisons with our findings challenging. On the other hand, our findings are, in general, in line with previously published studies on various experimental models, including to PPARα receptor reporter assays or knockout cell and animal models [21,22]. Importantly, our data are consistent with clinical observations of the lipid-lowering effects of mildly elevated serum BR concentrations, as well as negative associations between serum bilirubin concentrations and obesity and MASLD [17]. Our results contribute to the growing body of evidence supporting the potential metabolic benefits of BR, which deserves further investigation with an emphasis on more standardized and physiologically relevant conditions, together with designing mechanistic studies using biological material from patients with MASLD.

## Abbreviations

ANGPTL4: angiopoietin-like 4
BR: bilirubin
CTRL: control
CAR: constitutive androstane receptor
CPT1A: carnitine palmitoyltransferase 1
CVD: cardiovascular disease
DAPI: 4′,6-diamidino-2-phenylindole
DMSO: dimethyl sulfoxide
DMEM: Dulbecco’s Modified Eagle Medium
ET: electron transfer
FFA: free fatty acid
FCCP: carbonyl cyanide-p-trifluoromethoxyphenyl-hydrazone
FF: fenofibrate
FGF21: fibroblast growth factor 21
GC/MS: gas chromatography/mass spectrometry
HepG2: human hepatoblastoma cell line
LIN: linoleic acid
MASLD: metabolic dysfunction-associated steatotic liver disease
MTT: (3-(4,5-di methyl thiazol-2-yl)-2,5-diphenyltetrazolium bromide dye), OA, oleic acid
PA: palmitic acid
PDK4: pyruvate dehydrogenase kinase 4
PCR: polymerase chain reaction
PPARα: peroxisome proliferator-activated receptor α
PXR: pregnane X receptor
STEA: stearic acid
HPRT: hypoxanthine-guanine phosphoribosyl transferase
T2DM: type 2 diabetes mellitus
